# Metagenomic Hi-C of a Healthy Human Fecal Microbiome Transplant Donor

**DOI:** 10.1128/MRA.01523-19

**Published:** 2020-02-06

**Authors:** Matthew Z. DeMaere, Michael Y. Z. Liu, Enmoore Lin, Steven P. Djordjevic, Ian G. Charles, Paul Worden, Catherine M. Burke, Leigh G. Monahan, Melissa Gardiner, Thomas J. Borody, Aaron E. Darling

**Affiliations:** a The ithree institute, University of Technology Sydney, Sydney, NSW, Australia; b The Centre for Digestive Diseases, Five Dock, NSW, Australia; c The Quadram Institute, Norwich, United Kingdom; Georgia Institute of Technology

## Abstract

We report the availability of a high-quality metagenomic Hi-C data set generated from a fecal sample taken from a healthy fecal microbiome transplant donor subject. We report on basic features of the data to evaluate their quality.

## ANNOUNCEMENT

Metagenomic Hi-C is a recently emerged technique that enables the physical proximity of DNA sequences in a sample to be assayed (1–3). This type of three-dimensional (3D) spatial information about sequences has historically been missing from metagenomic shotgun sequencing data sets (4) and has led to the development of extensive, elaborate, and often failure-prone computational methods that attempt to reconstruct genomic content using other signals in the data (5).

We generated metagenomic Hi-C data for a human fecal sample as part of a larger technology evaluation program that also evaluated metagenomic double-digest restriction-site-associated DNA (ddRADseq) (6) and low-cost, low-bias Illumina shotgun library preparation protocols (7). The sample was obtained from a member of the healthy fecal microbiome transplant (FMT) donor pool used at the Centre for Digestive Diseases (Five Dock, NSW, Australia) in 2014. Briefly, the sample was collected fresh, stored frozen at –80°C for 1 year, and then thawed, cross-linked with 1% formalin for 1 hour, quenched with 125 mM glycine for 30 min, and stored frozen again prior to shipping to Phase Genomics LLC (Seattle, WA, USA) for Hi-C library preparation using an established protocol (8) and sequencing on an Illumina NextSeq 500 instrument. Ethical approval for this study was obtained from the University of Technology Sydney Human Research Ethics Committee (UTS HREC reference number 2014000448).

Sequencing produced 20.1 million 150-bp shotgun read pairs (totaling 5.8 Gbp) and 71.6 million 80-bp Hi-C read pairs (totaling 11.4 Gbp) composed of two technical replicates. The fraction of read pairs containing proximity ligation junctions (Hi-C read pairs) was estimated using the recently developed qc3C tool v0.2.6.6 (9) (default parameters used). qc3C has two methods for estimating the fraction of Hi-C read pairs in the data, (i) by mapping reads to a metagenome assembly and (ii) using an assembly-free technique based on k-mer counts. Using the mapping-based technique, the fraction of Hi-C read pairs was estimated to be within the range of 0.36 to 0.67%. To put this in context, the same estimate for another recently published metagenomic Hi-C data set (8) was 1.38 to 2.38%.

**FIG 1:**
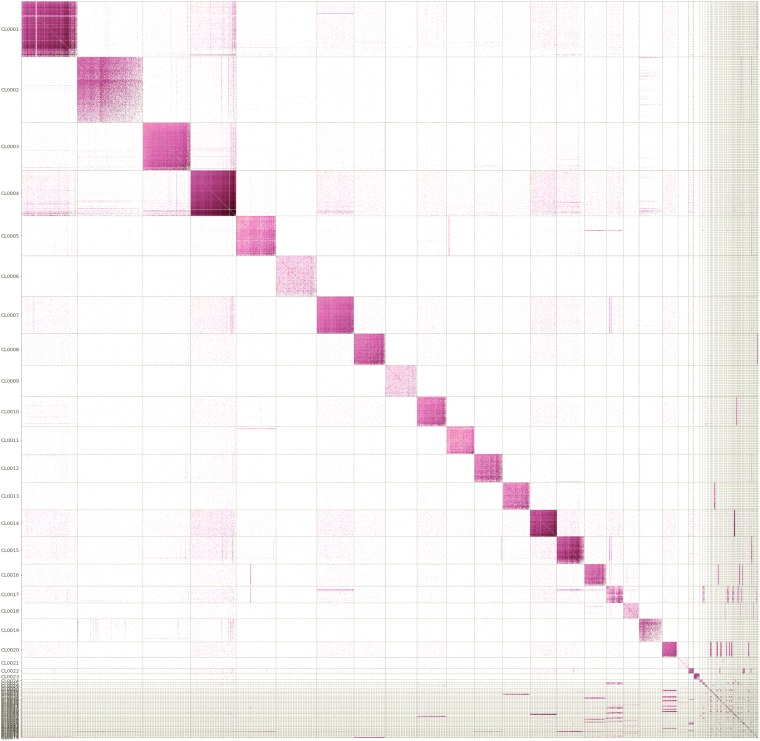
Hi-C contact map generated using bin3C from the metagenome assembly, ordered by decreasing cluster extent. Rows and columns correspond to contigs binned in windows of no more than 5 kbp. The log-scaled intensity of each cell represents the normalized interaction strength derived from the observed number of Hi-C read pairs that link the pair of loci. Blocks of color along the diagonal line correspond to groups of contigs that are in physical contact in the sample, typically because they are in the same chromosome or cell. Light dashed lines indicate the cluster boundaries determined with bin3C; the large bins correspond to MAGs.

Cleanup of the shotgun and Hi-C read sets was performed with fastp v0.20.0 (10) using default options, with the exception that overlapping shotgun pairs were merged. A metagenomic assembly was generated from the cleaned shotgun reads using SPAdes v3.13.1 (11) (command-line “–meta”) and comprised 181,642 contigs and a total size of 196,582,935 bp (*N*_50_, 2,965 bp). The scaffolds from this assembly were used in conjunction with the metagenomic Hi-C data from the same sample to reconstruct metagenome-assembled genomes (MAGs) using bin3C v0.3.3 (Fig. 1) (10) (command-line “cluster –min-signal 4 –n-iter 20 –seed 12345 –assembler spades”). The analysis yielded 15 genomes that were estimated to be ≥50% complete with ≤5% contamination as determined via CheckM v1.0.18 (12) (default parameters used).

The data we have released may be useful for a range of analyses, including the study of host-virus and host-plasmid associations, as well as the study of the 3D chromosome structure of dominant members of the human gut microbiome.

### Data availability.

Metagenomic Hi-C data are available under the Sequence Read Archive accession numbers SRR7427737 and SRR10566997. The corresponding shotgun library is available under accession number SRR5298275. The metagenomic assembly and derived metagenome-assembled genomes produced using bin3C are available from zenodo (https://zenodo.org/record/3598124).
